# Monitoring and tracking the spread of SARS-CoV-2 in Asturias, Spain

**DOI:** 10.1099/acmi.0.000573.v4

**Published:** 2023-09-27

**Authors:** Jose Maria Gonzalez-Alba, Susana Rojo-Alba, Zulema Perez-Martinez, Jose A. Boga, Marta Elena Alvarez-Arguelles, Juan Gomez, Pablo Herrero, Isabel Costales, Luz Maria Alba, Gabriel Martin-Rodriguez, Rainer Campo, Cristian Castelló-Abietar, Marta Sandoval, Fátima Abreu-Salinas, Eliecer Coto, Mercedes Rodriguez, Pablo Rubianes, Maria Luisa Sanchez, Fernando Vazquez, Luis Antuña, Victoria Álvarez, Santiago Melón García

**Affiliations:** ^1^​ Servicio de Microbiología, Oviedo, Spain; ^2^​ Instituto de Investigación Sanitaria del Principado de Asturias (ISPA), Oviedo, Spain; ^3^​ Servicio de Genética Molecular, Oviedo, Spain; ^4^​ Servicio de Urgencias, Hospital Universitario Central de Asturias, Oviedo, Spain

**Keywords:** SARS-CoV-2, variants, mutations, surveillance

## Abstract

Mutational analysis of severe acute respiratory syndrome coronavirus 2 (SARS-CoV-2) can quantify the relative importance of variants over time, enable dominant mutations to be identified, and facilitate near real-time detection, comparison and tracking of evolving variants. SARS-CoV-2 in Asturias, an autonomous community of Spain with a large ageing population, and high levels of migration and tourism, was monitored and tracked from the beginning of the pandemic in February 2020 until its decline and stabilization in August 2021, and samples were characterized using whole genomic sequencing and single nucleotide polymorphisms. Data held in the GISAID database were analysed to establish patterns in the appearance and persistence of SARS-CoV-2 strains. Only 138 non-synonymous mutations occurring in more than 1 % of the population with SARS-CoV-2 were found, identifying ten major variants worldwide (seven arose before January 2021), 19 regional and one local. In Asturias only 17 different variants were found. After vaccination, no further regional major variants were found. Only half of the defined variants circulated and no new variants were generated, indicating that infection control measures such as rapid diagnosis, isolation and vaccination were efficient.

## Data Summary

Data Availability in GISAID (https://www.gisaid.org/) and Supplementary Material. VOCs are defined in (https://www.ecdc.europa.eu/en/covid-19/variants-concern).

**Impact Statement**

The evolution of severe acute respiratory syndrome coronavirus 2 (SARS-CoV-2) has been a concern in the last 2 years. Changes that were most widespread in the population were sought, namely those that should cause some change in the phenotype or in the epidemiological characteristics. Before severe containment measures were imposed and only an incipient vaccination programme was in place, the epidemic reactivated in Asturias via variants that were spreading worldwide, although the region did not generate its own variants. Two further waves were observed, both of which involved few variants from distant geographical areas, and, again, the region did not develop its own variant. The Asturian model indicates that the movement of people is capable of taking any variant to any location, but infection control measures such as rapid diagnosis, isolation and vaccination could be capable of controlling the circulation of these variants. The SNP method allowed us to identify the most important variants circulating, thus showing it to be a good method of epidemiological control complementary to whole genome sequencing.

## Introduction

An unknown human coronavirus (hCov-19) was first detected in late 2019 in patients in Wuhan, China [1]. On 11 March 2020, the World Health Organization (WHO) declared a pandemic [2]. The first virus genomes and associated data were publicly shared online (https://www.gisaid.org/) on 10 January 2020 [3]

Since severe acute respiratory syndrome coronavirus 2 (SARS-CoV-2) is an RNA virus, genomic changes are quite frequent and continued genomic surveillance strategies are needed to improve both the monitoring of and our understanding of current epidemics. Characterization of the virus in real time allows us to know each variant’s transmission efficiency as well as the severity of the disease and thus make changes to patient management. Phylogenetic analysis of whole-genome multiple sequencing alignment is a good tool for this, but is time-consuming. Mutational analysis, on the other hand, can assess changes in viral genomic diversity, quantify the relative importance of variants over time, enable the dominant mutations to be identified, and facilitate near real-time detection, comparison and tracking of evolving SARS-CoV-2 variants. The large number of sequences available in GISAID also allows the rise of mutations found in different clades and in different countries to be highlighted and tracked. In this study, the SARS-CoV-2 epidemic in Asturias was monitored and tracked from its beginning in February 2020 until its decline and stabilization in August 2021. Asturias is an autonomous community in Spain with a large ageing population (in 2021, the population over 65 years of age comprised 24.4%, while in Spain as a whole it was 14.4%), and has high levels of migration and tourism (it received 4.5 % of its population through immigration and 145.5 % of its population through tourism, in the same year) [4–6]. In this context, it was able to maintain the incidence of SARS-CoV-2 cases at zero for 25 days in July 2020, in contrast to the rest of Spain. This was achieved with rapid diagnosis of infection, use of comprehensive control measures and high vaccination capacity. Data from the region were compared with those from other parts of the world to determine the evolution of SARS-CoV-2, and the influence of specific situations in Asturias in contrast to other countries.

## Methods

This study used a number of approaches. First, locally collected samples were characterized using either whole genomic sequencing (WGS) or single nucleotide polymorphisms (SNPs). In addition, data held in the GISAID database were analysed to establish patterns in the appearance and persistence of SARS-CoV-2 strains. The local data were then compared with the data from GISAID to track the evolution of the variants in Asturias compared to what was happening in another part of Spain (Madrid) as well as in Spain as a whole and with other countries in Europe and around the world.

### WGS method

SARS-CoV-2 whole genomic sequences were obtained on 708 nasopharyngeal swabs collected in Asturias between March 2020 and July 2021 from 30 904 patients in whom the virus was detected (15 949 after January 2021), by using an Ion Torrent platform [7]. Briefly, cDNA was synthesized from 10 μl of RNA extract using the SuperScript VILO cDNA synthesis kit (Invitrogen) in accordance with the manufacturer’s instructions. The Ion AmpliSeq SARS-CoV-2 research panel, supplied for this study by Thermo Fisher Scientific, contained two pools, one with 250 primer pairs and the other with 244, designed to respectively cover the SARS-CoV-2 genome with 125 and 275 bp overlapping amplicons. Samples were amplified for 20 cycles with a 4 min extension time. Libraries were prepared on the Ion Chef system following the instructions of the user guide. Amplified samples were then sequenced, using Ion 540 chips (Thermo Fisher Scientific), with the Ion S5 system (Thermo Fisher Scientific), following the instructions set out in the manufacturer’s user guide. Initially the samples were chosen randomly and later (from November 2020 onwards) variants of interest were specifically selected. Sequences were uploaded to the GISAID database.

### SARS-CoV-2 PCR method

Between January and July 2021, 13 229 samples from Asturias were analysed using SNPs. Initially only the S gene in position 501 was studied, but from March 2021 onwards, in line with the developing information being released by the WHO, positions 417, 452, 478 and 484 were also assayed for SNPs using sequence-specific forward and reverse oligonucleotide primers, along with TaqMan MGB variant-specific probes, each with a different reporter dye at the 5′ end and a non-fluorescent quencher (NFQ) at the 3′ end. The design of the primers and probes, as well as reaction and amplification protocols, was carried out following Sandoval-Torrientes [8].

### Analysis of the cGISAID database

From the GISAID database [9], a total of 3 116 331 genome sequences had been used as of 31 August 2021, via gisaid.org/EPI_SET_230811vf. The sequences were recorded together with the country of origin and the date of collection. While there may be anomalies in terms of the frequency of occurrence of the sequences uploaded in GISAID, it must be acknowledged that during the pandemic, such imperfections and inconsistencies had to be tolerated to avoid losing potentially important samples and reducing the global panorama.

The genome sequences were aligned with the reference genome of SARS-CoV-2 (NC_045512.3) by MAFFT (Multiple Sequence Alignment Software Version 7). Each of the coding regions (E, M, N, ORF1a, RDPD, ORF1ab, ORF3a, ORF6, ORF7a, ORF7b, ORF8, ORF10, S) was extracted separately from the alignments and nucleotides in the coding regions were converted to their corresponding encoded amino acid residues (SeaView v.4). SNPs were retrieved from the aligned regions in line with the reference genome. To avoid loss of mutations in non-sequenced fragments, we have not made any selection to include all possible mutations. Non-synonymous mutations with a frequency of more than 1 % were used in subsequent analyses. The data were used to visualize the patterns occurring each month by calculating the relative abundance of each mutation (number of strains with a specific mutation/total number of strains). On the basis of this, worldwide major variants (WMVs) were identified, along with their related strains, namely strains sharing the same mutation pattern. In naming the strains for the purpose of this study, each variant was given a number indicating the temporal order of its appearance (i.e. WMV1 appeared before WMV2) and those which appeared at the same time were given lower case letters to distinguish them.

The geographical origin of each sequence was noted and used to visualize the temporal patterns in different countries and in this way the major variants dominating the epidemic at the regional (country) level were defined (RMVs). In addition, the data for each autonomous community in Spain, where more than 500 sequences were identified, were used to define major variants that dominated the epidemic at the local (Spain) level (LMVs). Only variants that appeared in more than 5 % of the population testing positive for SARS-CoV-2 in the corresponding area were taken into account in the analyses. Once the variants were identified, we considered the simplest evolutionary hypothesis to be that each variant had originated through gaining one or more mutations compared to the previously identified major variant (ancestor) whose mutations it shared. Each variant is defined by a specific pattern of mutations so corresponds to multiple lineages as defined by pangolin (cov-lineages.org). Here, for simplicity, each defined variant is identified with the pangolin lineage present in the highest percentage. As the pandemic progressed, the WHO began to identify variants of concern (VOCs), defined by the pattern of mutations in their S gene [10], which correspond to the major variants (WMV, RMV and LMV) discussed here. The evolution of the epidemic in Asturias was compared with that in another autonomous community in Spain (Madrid), with Spain as a whole, with neighbouring countries (France and Portugal), with countries that are representative of other areas of the world (England, Germany and Italy from Europe, Russia from Eastern Europe, Israel from the Middle East, India from Asia, Brazil from South America, the USA from North America, Australia from Oceania, South Africa from Africa) and with the global statistics. The number of sequences analysed for each area is shown in Table S1, available in the online version of this article.

## Results

### Identification of mutations and major variants using GISAID data

**Fig. 1.**
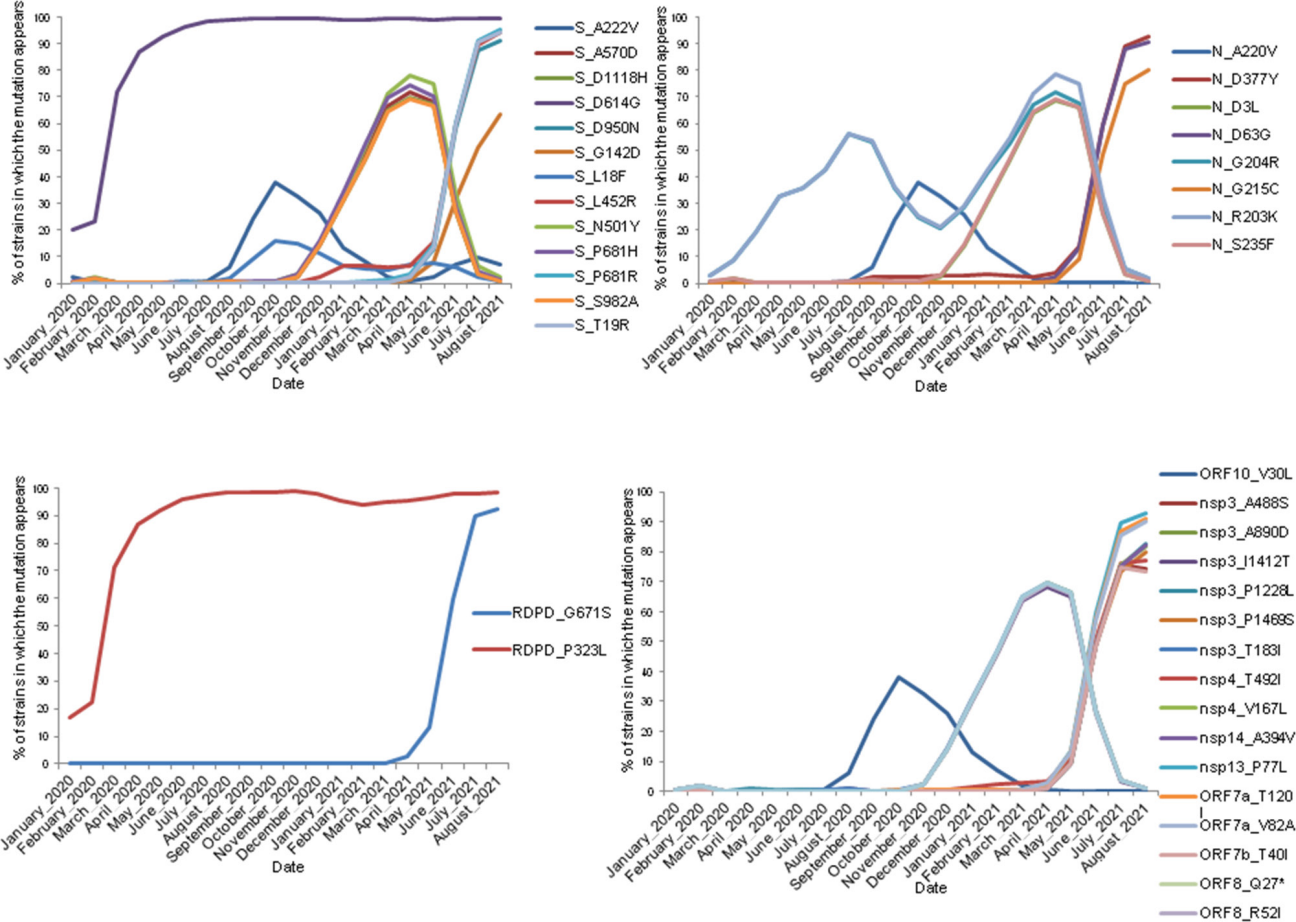
Evolution of the major mutations during the waves of SARS-CoV-2 worldwide by genes using GISAID data. The *y*-axis indicates the percentage of strains in which the mutation appeared worldwide each month (January 2020 to August 2021).

In the >3×10^6^ of sequences analysed after 18 months of the pandemic, 138 amino acid mutations that appeared in more than 1 % of the world viral population were found: 40 in the gene S, 36 in ORF1a, 18 in the N gene, 12 in ORF1ab, 9 in ORF3a and ORF8, 7 in RDPD, 3 in ORF7a and 1 each in ORF10, ORF7b, the E gene and the M gene. Of these 138 mutations, 46 (14 gene S, 10 ORF1a, 8 gene N, 2 ORF1ab, 2 ORF3a, 4 ORF8, 2 RDPD, 2 ORF7a, 1 ORF7b and 1 gene M) were found in more than 10 % of worldwide viral strains and 13 of them in 5–10 % (Table S2). The average number of mutations in the genome of the SARS-CoV2 data analysed was 20±8 (range: 0–99); in Asturias this was 19±8 (range: 1–57) (Fig. S1). The evolution over time of the main mutations during the different epidemic waves worldwide is shown in Fig. 1 (Table S3).

A total of 30 major variants were identified during the 18 months of the study: 10 were WMVs, 19 were RMVs identified at the country level, and one was an LMV identified from Spain (Table 1).

**Table 1.** SARS-CoV-2 major variants (MVs) identified using GISAID data

Variant	Lineage	VOC	Mutations shared	Mutations gained											
				S	RDPD	M	N	E	ORF1a	ORF1ab	ORF3a	ORF7a	ORF7b	ORF8	ORF10
V0															
WMV1a	B.1			D614G	P323L										
WMV1b	B.1		WMV1a				R203K G204R								
WMV2a	B.1.117		WMV1a	A222V			A220V								V30L
WMV2b	B.1.117		WMV2a	L18F											
WMV3b	B.1.1.7	Alpha	WMV1a	P681H S982A A570D N501Y D1118H T716I			S235F R203K D3L		A1708D I2230T T1001I					Y73C R52I Q27*	
WMV3c	B.1.1.7	Alpha	WMV3a	K68*											
RMV9c	B.1.1.7	Alpha	WMV1b	P681H A570D T716I N501Y											
WMV4a	B.1.617.2	Delta	WMV1a	L452R P681R T478K			D377Y			P77L					
WMV4b	B.1.617.2	Delta	WMV4a	D950N T19R G142D	G671S D950N		D63G G215C		T3255I P2287S V2930L A1306S P2046L	A995V		T120I V82A	T40I		
WMV4c	B.1.617.2	Delta	WMV4b	T95I											
RMV1a	P.1	Gamma	WMV1b	V1176F											
RMV8	P.1	Gamma	RMV1a	D138Y E484K H655Y K417T P26S T1027I R190S L18F T20N N501Y			P80R		S1188L K1795Q	E341D					
RMV1b	D.2		WMV1b	S477N											
RMV2a	B.1.1.141		WMV1b	M153T											
RMV2b	A.2.5			L452R			P383L M234I S197L P365S		T3255I H3580Q K1657E F3071Y L4F	P77L	G196V S74F			L84S	
RMV3a	B.1.351	Beta	WMV1a	D80A E484K A701V N501Y K417N D215G					K1655N						
RMV11	B.1.351	Beta		E484K A701V N501Y											
RMV3b	B.1.497		WMV1a						S3884L T265I	Q1480L	Q57H				
RMV4a	B.1.2		WMV1a				P67S P199L		L3352F	N730D	G172V				
RMV4b	A.2.5			L452R			M234I S197L		T3255I H3580Q L4F F3071Y	P77L	G196V S74F			L84S	
RMV5	B.1.427		WMV1a	L452R S13I W152C						D260Y					
RMV6	B.1.258.17		WMV1a		V720I;S N439K				M4241I I2501T	H290Y A598S					
RMV7a	B.1.524		WMV1a	A701V			S194L		T2791I T2016I T3287A	L428F					
RMV7b	B1.621	Mu	WMV1a	R346K					T1055A T1538I	P419S					
RMV7c	B.1.617.1	Kappa	WMV1a	L452R P681R E484Q											
RMV9a	B.1.1.317		WMV1b	D138Y Q675R A845S S477N			A211V		D589G						
RMV9b	C.37	Lambda	WMV1b	L452Q F490S											
RMV10	B.1.525	Eta		Q677H E484K F888L			A12G	L21F	T2007I						
LMV	B.1.575.1		WMV1a	P681H T716I S494P		I82T	T205I A414S		T3255I T3750I T265I	P47S	Q57H				

The first letter indicates the geographical area in which they have major spread, worldwide (W), in a country (R) or in a region of a country (L). The number indicates the chronological order in which they were identified in the same geographical area. A lowercase letter indicates variants identified at the same time and in the same area. In the table, PANGO and VOC classification, the mutations that define the variants and their possible precursor are indicated.

At the beginning of the pandemic, in January and February 2020, the V0 variant (>80 %) predominated around the world. In March 2020, however, WMV1a/WMV1b (B.1 lineages and sub-lineages) accounted for 70 % of infections worldwide, rising to 90 % in June. Between July and November 2020 these variants gained mutations and shared a niche with WMV2a, WMV2b (B.1.117) and RMV1b (identified in Australia), and RMV4a (identified in USA). In December, WMV3a, WMV3b and WMV3c variants (B.1.1.7/alpha) appeared and were the most common variants in circulation until May 2021 (59%). During this period WMV2a/WMV2b were displaced and shared a niche with RMV5 (identified in USA) and RMV8 (gamma, identified in Brazil). However, in May 2021 new variants appeared (WMV4a, WMV4b and WMV4c) that belong to the B.1.167.2 lineage and became known as VOC delta displacing the rest of the variants (88 % in August 2021).

**Fig. 2.**
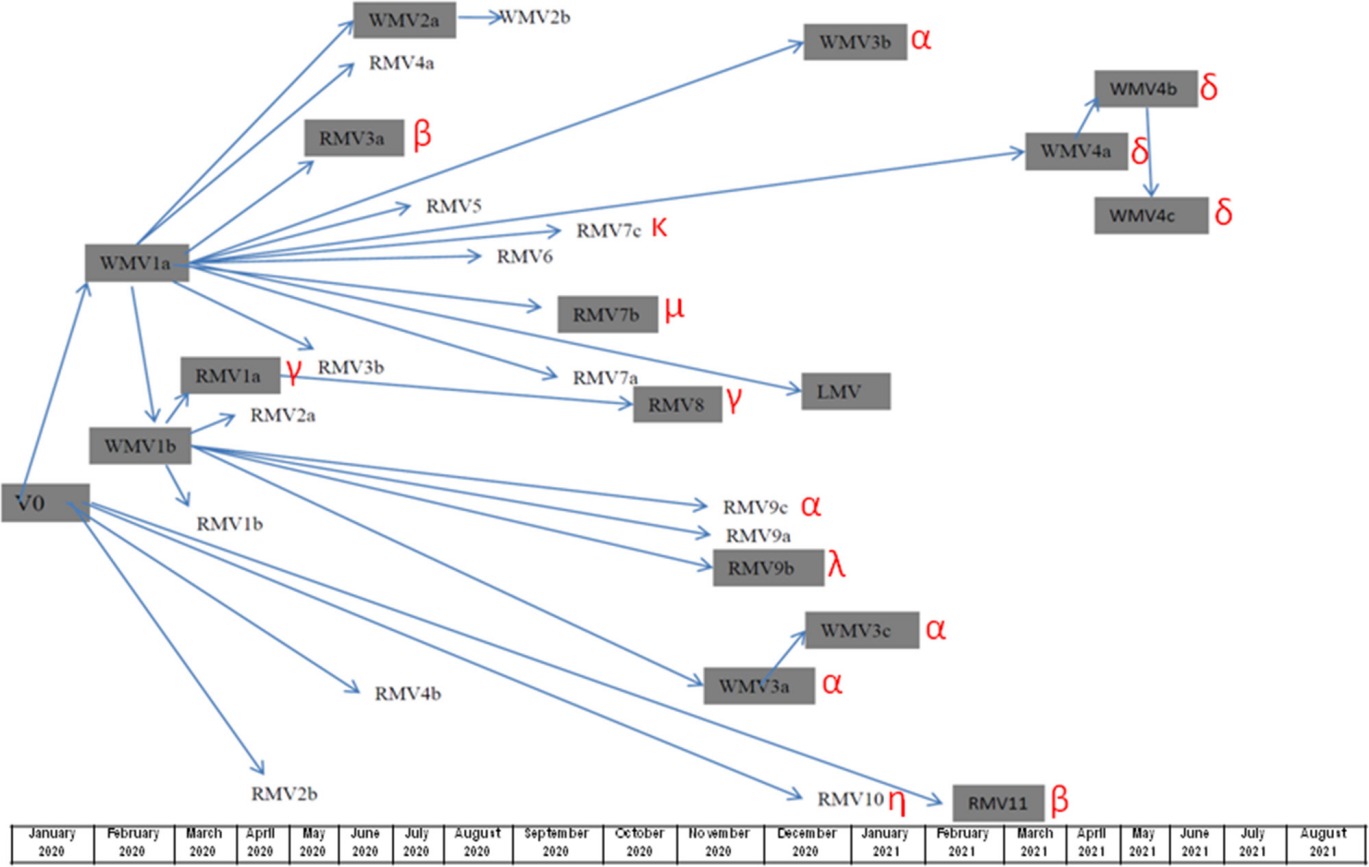
Estimation of the temporal evolution of the variants according to their first appearance and the mutations they share with the existing variants, using GISAID data. Asturias variants are in black. Greek symbols for VOC classification have been added.

The distribution over time of the major variants that had a worldwide prevalence in any month of 5 % is shown in Table 2 (all in Table S4). The evolution of variants worldwide is shown in Fig. 2.

**Table 2.** Temporal distribution of those major variants that represented more than 5 % of the viral population worldwide in a given month using GISAID data The number indicates the percentage in that month.

			2020												2021							
**Variant**			**January**	**February**	**March**	**April**	**May**	**June**	**July**	**August**	**September**	**October**	**November**	**December**	**January**	**February**	**March**	**April**	**May**	**June**	**July**	**August**
V0			97	80	29	15	9	6	4	3	3	3	3	3	6	7	5	6	6	3	4	4
WMV1a			2	13	52	54	56	50	38	35	33	29	32	27	22	18	12	11	8	7	7	8
WMV1b	B.1			6	18	31	34	40	36	40	32	23	17	13	10	8	7	6	5	2		
WMV2a	B.1.117								1	5	15	22	18	16	9	5	1					
WMV2b										1	9	15	14	10	4	2	1					
WMV3a	B.1.1.7	Alpha											2	11	23	30	38	37	33	13	2	
WMV3b																2	4	6	7	3		
WMV3c														2	5	9	15	19	19	7	1	
WMV4a	B.1.617.2	Delta																2	7	33	48	46
WMV4b																			1	7	16	17
WMV4c																			4	16	21	25
RMV8	P.1	Gamma														1	2	4	5	4	1	
RMV1b	D.2							1	16	9	1											
RMV4a	B.1.2							2	3	3	4	5	11	11	11	8	4	1				
RMV5	B.1.427													2	5	4	4	2	1			

### The SARS-CoV-2 pandemic in Asturias

On the basis of WGS data, it can be seen that 25 of the sequences corresponded to the ancestral variant (V0), 602 sequences to one of nine WMVs, 77 sequences to one of seven RMVs, and four sequences to the single LMV identified in Madrid. Of these variants, 450 belonged to VOC alpha, beta, gamma, delta, lambda and mu (Table 3). In Madrid the same variants circulated plus WMV2b, RMV1b (D.2 identified in Australia), RMV2a (B.1.1.141 identified in Russia), RMV4a (B.1.2 identified in USA), RMV5 (B.1.427 identified in USA) and RMV10 (B.1.525 identified in Nigeria). In Spain three more RMVs were found than in Madrid: RMV2b (A.2.5 identified in Panama), RMV6 (B.1.258.17 identified in Croatia) and RMV7c (B.1.617.1, kappa identified in India). In the rest of Europe, RMV4b (A.2.5 identified in Panama) and RMV9a (B.1.1.317 identified in Russia) were also found. In the rest of the world, RMV3b (B.1.497 identified in Korea) and RMV7a (B.1.524 identified in Malaysia) were also found. In South Africa, fewer major variants (16) were found than in Asturias (Table 4).

**Table 3.** Number of samples sequenced by WGS in Asturias The waves of the SARS-Cov-2 epidemic peaks in Asturias from 2020 to 2021 are indicated in red.

		2020										2021								
**Variant**	**VOC**	**March**	**April**	**May**	**June**	**July**	**August**	**September**	**October**	**November**	**December**	**January**	**February**	**March**	**April**	**May**	**June**	**July**	**August**	**Total**
V0		11	1											1	3	1	1	3	4	25
WMV1a		38	19	8								2	10	2	1	6	1		3	90
WMV1b		3										3	3	6	2	1				18
WMV2a										1	1	35	65	16	3					121
WMV3a	Alpha																2			2
WMV3b	Alpha											14	21	25	32	19	28	3	1	143
WMV3c	Alpha										2	20	39	41	36	29	5			172
RMV9c	Alpha												1	3		1	1			6
WMV4a	Delta																28	2	10	40
WMV4b	Delta																8	1	3	12
WMV4c	Delta																3	1		4
RMV1a	Gamma													1						1
RMV8	Gamma													6	17	9	9			41
RMV3a	Beta																6	1		7
RMV11	Beta														2					2
RMV7b	Mu															1	2	1	4	8
RMV9b	Lambda														2	3	4	3		12
LMV													4							4
Total		52	20	8	0	0	0	0	0	1	3	74	143	101	98	70	98	15	25	708

**Table 4.** Variants found in each studied area using GISAID data The number indicates the chronological order in which they were detected in each zone. The Total numbers indicate the number of different variants identified in each zone.

	Asturias	Madrid	Spain	England	Germany	France	Portugal	Italy	Russia	Israel	India	Brazil	USA	Australia	South_Africa
V0	1	1	1	1	1	1	1	1	2	1	2	1	1	1	1
WMV1a	1	1	1	1	1	1	1	1	1	1	1	1	1	1	1
WMV1b	1	1	1	1	1	1	1	1	1	1	1	1	1	1	1
WMV2a	2	1	1	1	2	2	2	2	5	2	7	5	4	3	
WMV2b		2	3	3	3	3	2	2	6	2	7		5	5	
WMV3a	8	6	7	9	7	6	4	5	6	3	8		8	9	9
WMV3b	3	3	1	6	6	4	2	3	5	2	5	4	5	7	5
WMV3c	2	3	6	7	7	7	3	4	5	2	5	5	5	7	6
RMV9c	4	2	4	6	7	5	3	4	6	2	7	5	5	6	5
WMV4a	8	8	10	4	10	10	6	5	8	4	4	8	7	11	7
WMV4b	8	8	11	11	9	10	6	5	8	6	8	8	6	11	7
WMV4c	8	9	10	8	11	9	7	5	8	6	9	9	10	9	8
RMV1a	5	3	6	3	8	8	2	4	4	3	3	1	3	1	2
RMV8	5	4	7	10	9	8	5	5		4		2	6	9	
RMV1b		3	6	2	5	3	3	6	4	2	4	3	5	1	4
RMV2a		4	6	5	6	6			2	2	8	2	6	5	
RMV2b			8	10	10		4	5					6	8	
RMV3a	8	5	6	9	7	7	4	5	5	2	7	7	7	7	3
RMV11	6	7	8	9	7	8	5	5	7	2	8	7	7	8	3
RMV3b													9		
RMV4a		3	2	4	8	8	4	6		2	6	5	2	2	
RMV4b					8			6					7	8	
RMV5		4	7	9	9	8		5		2			4	7	
RMV6			5	5	3	3	3	2	8	2	7		4	4	
RMV7a											7			7	
RMV7b	7	6	9	12	11	10	7	8				9	9		
RMV7c			10	10	10	9	6	7	8	6	4		7	10	8
RMV9a				7	4			7	3	2			6	9	
RMV9b	6	6	9	10	9	10	6	9		5		6	7	11	
RMV10		5	7	9	8	8	6	5	9	3	8	6	6	9	6
LMV	4	3	6	10	9		6	7					10		
Total	18	24	27	28	29	25	25	28	21	25	22	20	30	28	16

**Fig. 3.**
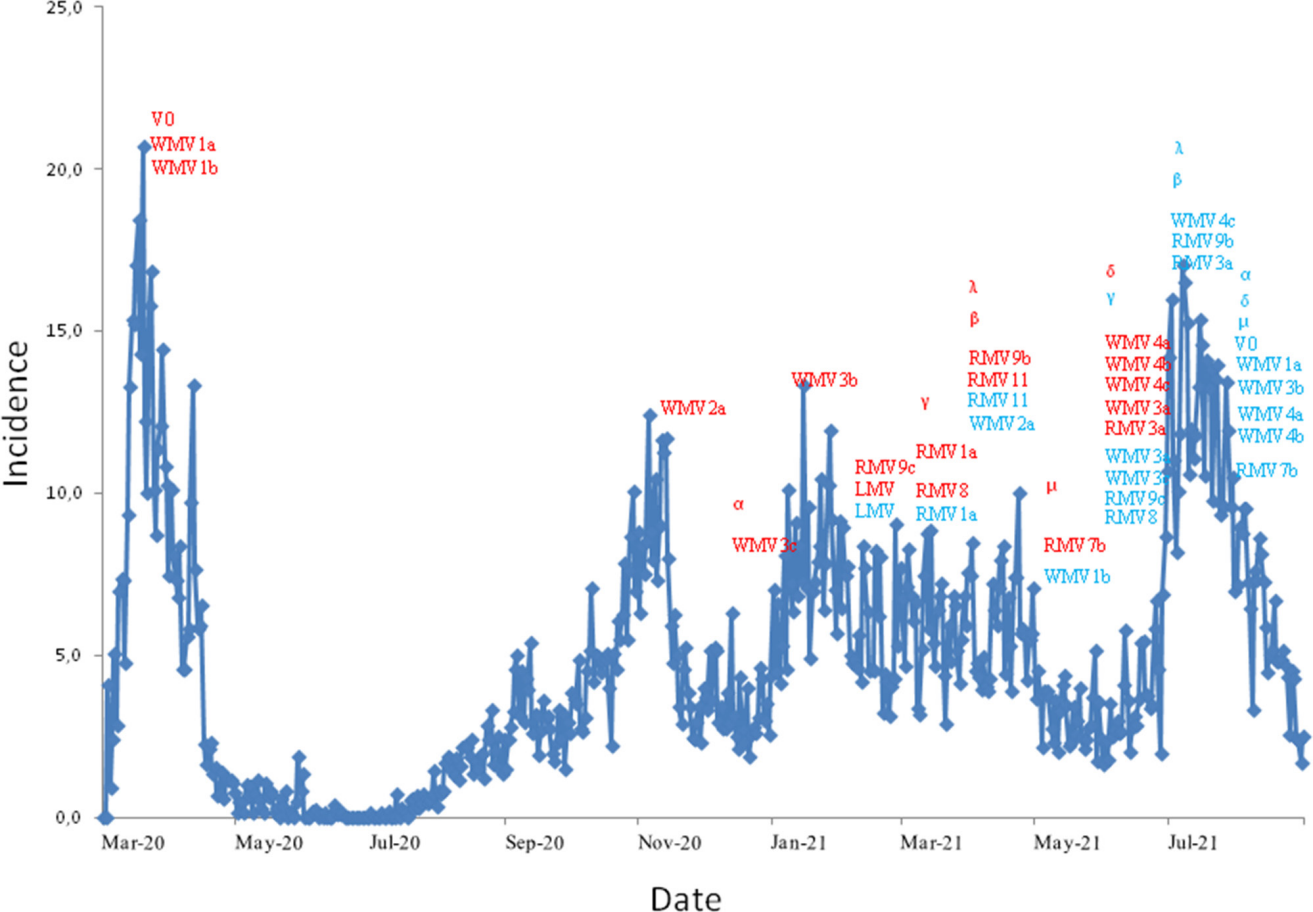
SARS-CoV-2 epidemic waves in Asturias, from February 2020 to August 2021. Red indicates when each variant was first sequenced and blue the last time.

Asturias experienced four SARS-CoV-2 waves between February 2020 and August 2021 (Fig. 3). In the period between March 2020 and October 2020, the first wave of the epidemic occurred in Asturias, characterized by the presence of multiple variants without the D614G spike mutation (21 % V0), as well as variants WMV1a and WMV1b (79 %) followed by a period of epidemic control. During this period, in Spain WM2a appeared (35 % in June) and spread to the rest of Europe except Portugal and Russia. In other parts of the world, their own variants also spread, RMV3a (B.1.351, beta, 5 % in September) and RMV11 (B.1.351, beta, 11 % in September) in South Africa, RMV1a (P.1, gamma, 31 % in March) in Brazil, RMV4a (B.1.2, 8 % in July) in the USA and RMV1b (D.2, 7 % in May) in Australia.

In the second wave of November 2020, WMV2a was found for the first time, as was the case in France and Australia. WMV3a (alpha) and RMV9c (alpha, identified in Austria) emerged in England. In December 2020, with the epidemic apparently under control, WM3a was detected.

Between January 2021 and May 2021 (third wave), initially WMV2a was the dominant variant and was displaced by WMV3b, WMV3c (B.1.1.7, alpha) and RMV8 (P.1, gamma) identified in Brazil. During this period an LMV (a local variant identified in Madrid in February that quickly disappeared) and multiple VOCs, namely RMV9b (C.37, lambda) identified in Russia although in no month was it found in >5 %, RMV11 (B.1.351, beta) identified in Cuba but of the countries studied, was only found in >5 % in South Africa, and RMV7b (B.1.621, mu) identified in Colombia, were circulating. In April 2021, WM4a (delta) appeared in India and in June, with the epidemic apparently under control, WM4 (B.1.617.2, delta) already spreading throughout Spain and RMV3a (B.1.351, beta) identified in South Africa were detected in Asturias. RMV3a was only found in >5 % in Spain in July 2021.

In July 2021 (fourth wave) five worldwide and three regional variants were found. By August 2021, the delta variant represented 52 % of strains isolated and dominated the epidemic as in the rest of the world (Table 3). The variation found between the countries studied is detailed in Table 4, Fig. S2 and Table S5.

Using SNP data, we found 11 423 sequences that belonged to VOCs (alpha, beta, gamma, delta) and 1906 from other lineages. In the third wave, alpha represented 95 % of strains and by August 2021 the delta variant represented 87 % (Table 5).

**Table 5.** Percentage of VOCs found in Asturias and total number of strains analysed each month (SNP/NGS)

		2020										2021							
		**March**	**April**	**May**	**June**	**July**	**August**	**September**	**October**	**November**	**December**	**January**	**February**	**March**	**April**	**May**	**June**	**July**	**August**
SNP	Alpha	0	0	0						0	0	28	70	97	96	94	80	45	11
	Beta	0	0	0						0	0	0	0	0	0	0	1	3	1
	Gamma	0	0	0						0	0	0	0	0	3	4	7	0	0
	Delta	0	0	0						0	0	0	0	0	0	0	6	48	87
	Others	0	0	0						0	0	72	30	3	1	2	6	4	1
	No. of strains (Total)	0	0	0						0	0	1463	1840	1627	1168	799	690	3852	1890
NGS	Alpha	0	0	0						0	67	46	42	65	69	69	36	20	4
	Beta	0	0	0						0	0	0	0	0	0	0	6	7	0
	Gamma	0	0	0						0	0	0	0	7	17	13	9	0	0
	Delta	0	0	0						0	0	0	0	0	0	0	40	27	52
	Others	100	100	100						100	33	54	57	25	9	11	2	20	28
	No. of strains (Total)	52	20	8						1	3	74	143	101	98	70	98	15	25

## Discussion

Genomic information for SARS-CoV-2 is essential to gain a better understanding of host–pathogen interactions, management of infection, and to design drugs and vaccines. It also enables the management of the pandemic on both a global and local level [11–17]. A surprising aspect of the SARS-CoV-2 pandemic was the unprecedented availability of scientific and technological resources that allowed access to the virus’s genomic characterization [18–20]. In this respect, the more complete but laborious WGS method and the faster SNP method have been added to the classical Sanger method of sequencing [21]. Phylogenetic methods are the gold standard for mapping viral outbreaks, assigning lineages, and tracing transmission of lineages that may be associated with increased transmission, disease severity or vaccine failure. The relatively low level of genetic diversity of SARS-CoV-2 precludes defining lineages using only the presence or absence of nucleotide changes at a subset of variable sites. WGS is necessary to confirm direct transmission and/or reinfection and to determine minority variants. Defining the different lineages and their evolutionary relationship makes it possible to identify mutations due to some selective advantage, such as escape from vaccines or greater transmissibility. However, the large number of available sequences poses a considerable computational challenge. The highly frequent SNP mutations could be associated with biological and clinical differences of the virus. The SNP approach can be used for epidemiological studies with large amounts of data without the need for large resources. The detection of mutations associated with high transmissibility or virulence will allow the generation of low-cost and rapid genotyping methods to identify variants of interest.

In this study we focus on data from Asturias, a region of Spain, one of the countries where the virus had serious impacts from the very beginning of the pandemic [22]. The data were analysed with our own system, as explained in the Methods section, to simplify evaluation of the results. This method allows the evolution of major variants of the virus to be understood and compared with that from different geographical areas around the world.

SARS-CoV-2 is defined by a small number of mutations in its genome. Analysing the GISAID database up to August 2021 (more than 3×10^6^ sequences), only 138 non-synonymous mutations occurring in more than 1 % of the population were found, these being mutations that may confer a selective advantage to the variant. The S gene was the most variable, with 40 non-synonymous mutations, of which just 17 were found in more than 5 % of the strains analysed. The accumulation of the spike mutation could potentially be affecting the stability of the spike and may therefore influence the virus’s binding affinity with the ACE2 receptor, and could therefore have an influence in terms of natural or triggered immunity [23]. Recently, a new variant (omicron) has shown up to 30 differents mutations in the S protein [17]. However, the role of spike mutations remains unclear [24].The largest gene, ORF1ab, which contains several overlapping ORFs (nsp1–nsp12), showed 36 possible changes (only 11 appeared in more than 5 % of strains). Finally, another structural gene (N) presented 18 different mutations, eight of them being present in more than 5 % of the strains analysed. The remaining SARS-CoV-2 genes showed fewer mutations. In Asturias, the strains sequenced showed an average of 19 changes, although some had up to 57 mutations. Similarly, in the GISAID sequences analysed, the average was 20 mutations. That said, as would be expected, SARS-CoV-2 strains continue gaining mutations over time, with 30 having been identified by July 2021.

A combination of 41 mutations defined 30 major variants that circulated and generated the various waves of the pandemic (ten throughout the world, 19 in certain countries and one only in a local area). A possible relationship between major variants can be established based on their amino acid mutations and the date they were first found. The changes that were most widespread in the population were sought, as they are the ones that should cause some change in the phenotype or in the epidemiological characteristics. These variants could have evolved from the D614 ancestral strain. From the ancestral strain V0, S_D614G and nsp12_P323L (present in WMV1a and WMV1b variants) appear to have had a selective advantage that enabled them to dominate the epidemic since May 2020. The earliest examples of sequences carrying the D614G mutation were found in China and Germany in late January 2020 [25]. This may be the ancestral variant which then acquired the mutation P323L, required for polymerase activity and predicted to diminish the efficacy of antiviral drugs [26, 27]. Four months later (June 2020), a new variant, WMV2, appeared in Spain and then later spread worldwide, accumulating four more mutations than WMV1a. These variants share a niche with the variants of the first worldwide wave and with regional waves in Russia (RMV2a), Australia (RMV1b) and Brazil (RMV1a, gamma), that accumulated a mutation with respect to WMV1b. The mutations from June to October 2020 did not endure so had little functional impact on SARS-CoV-2 infection [28–31]. During the same period, the accumulation of mutations in WMV1a generated eight more variants that gave rise to regional epidemic waves in the USA (RMV4a, RMV5), South Africa (RMV3a-beta), Brazil (RMV8-gamma), Malaysia (RMV7a), Croatia (RMV6), Colombia (RMV7b-mu) and India (RMV7c-kappa). In November 2021, a new variant, WMV3a (alpha), appeared in England from WM1b [32, 33], with potentially enhancing host cell fusion mechanisms facilitating cell entry [34, 35]. The accumulation of mutations in WMV1b generated six other variants that gave rise to regional epidemic waves in Russia (RMV2a, RMV9a), Brazil (RMV1a-gamma), Australia (RMV1b), Argentina (RMV9b-lambda) and Austria (RMV9c-alpha). Four months later (March 2021), a new variant, WMV4a (delta), appeared in India and spread worldwide, accumulating mutations from WMV1a to facilitate evasion of neutralizing antibodies, to enhance IL-8 expression or to play a role in regulating multiple innate immune pathways [36–40]. From WM4a two variants were generated, accumulating 17 mutations which accounted for 88 % of infections in the world in August 2021. The number of variants that make up the majority in just some countries is greater than the number of variants that later spread worldwide, and even variants that originated in one country but represent the majority in another can be found (RMV11 was the most common variant in Cuba but first appeared in South Africa).

In this study it was possible to verify that multiple major variants emerged before January 2021, when vaccination began. Subsequently, only four new major variants (three delta lineages and one beta lineage) emerged in March–April 2021. In addition, after vaccination, no further regional major variants were found. The measures adopted worldwide could have contributed to these results: less transmission and fewer circulating variants. In Asturias, over these 18 months, besides the V0 ancestral variant, only nine WMVs and eight RMVs, as well as one LMV were found. However, in most parts of the world, 30 different variants were in circulation, of which 14 emerged between April and October 2021.

The first case of SARS-CoV-2 found in Asturias was on 29 February 2020 (first wave February to October 2020). Since then, and up to the end of the study period, the WMV1a and WMV1b variants circulated, and they were responsible for the first Asturian epidemiological wave, which occurred at the same time as in the rest of the world. WMV1b circulated from the beginning of the epidemic as with worldwide, except in Brazil and Australia where the variants generated were RMV1a and RMV1b, respectively [41–43].

From May to October 2020, incidence in Asturias decreased to <5 %. Over a 25 day period between June and July, no new strains were isolated. This could have been due to rapid diagnosis and a rapid contingency action. While the epidemic, at this point, was under control, it continued to spread in the rest of the world.

July–August 2020, when containment measures were relaxed, coincided with the beginning of a second wave in Madrid due to WMV2a (lineage B.1.177), a variant first found in Spain in June 2020, but which spread to the rest of Europe probably due to international summer tourism [44]. The WMV2a variant was first found in Asturias in November 2020 (second wave November–December 2020), when the area entered its second wave, which had the highest incidence of infections during the study period. In this same period, the WMN3a variant (alpha) originated in England, arriving in Asturias at the end of 2020. At this point, it is worth noting the spread in Russia of its own RMV2a and in the USA of its own RMV4a. In Australia, RMV4a replaced RMV1b as the dominant strain. Particular mention should also be made of the situation in South Africa, where, since September 2020 it has had two of its own beta variants (RMV3a, RMV11). These variants share the mutation N501Y and are more lethal than the alpha variant, possibly because they also acquired a mutation at E484K [45]. Between September and December 2020, this high incidence may explain the high replication rate of the virus and, therefore, the production of multiple variants. These emerging strains compete for an ecological niche and, eventually, when conditions begin to disadvantage transmission rates (e.g. restrictions on contact between people, quarantine, physical distancing and compulsory mask wearing), one becomes the dominant strain.

The third wave in Asturias (January–May 2021) coincided with the start of the vaccination programme. During this period, WMV2a was displaced by WMV3 (WMV3a/WMV3c-alpha) and RMV8 (gamma). In Madrid and the rest of Spain, another regional alpha variant (RMV9c) dominated, which could be due to the introduction of variants from other European areas. In the rest of Europe there were multiple waves of increased infection also happening at this time. In Russia, however, there was no WMV2 and its own variants (RMV4a and RMV5) dominated. France had the highest number of different variants, coinciding with the epidemiological peak (900 cases/100 000 inhabitants in a 14 day period). In England only WMV3 was present at >5 %, and it spread throughout the rest of the world, although it was replaced by the WMV4 variant in May 2021. India had its own variant (RMV7c-kappa), but WMV4a became the dominant variant in March 2021. Brazil and South Africa continued with their own strains (gamma and beta, respectively). Australia, like France, had a large number of variants even though the epidemic was considered to be under control. In the USA, the data show how its own variants were displaced by WMV3 and RMV8 at the point where the peak of the epidemic was reached (1000 cases/100 000 inhabitants in a 14 day period) [46].

We can see that during this period, the behaviour of the variants was different in each country. Although in some (England, Spain and Germany) alpha was predominant, in others (France, Italy and Portugal) it coexisted with other variants, while it did not arrive at all in some countries (Russia). Eventually, in other countries (India, Israel, Brazil, USA, South Africa and Australia) alpha began to circulate alongside their own variants. Interestingly, this behaviour appears to have depended more on the control measures taken in a particular country rather than geographical proximity.

During this period, with the idea of quickly determining the variants circulating in Asturias, and because several variants sharing the same mutation were present (alpha, beta and gamma VOCs), the SNP method was developed. It was later extended to include a greater number of positions in order to better classify the increasing number of strains circulating in Asturias. The greater number of sequences analysed using this method allowed us to assess the epidemic more accurately and enabled us to identify that the epidemic consisted of B.1.177 (28 %) and alpha (72%) strains until April 2021, at which point gamma appeared (3%). B.1.177 then disappeared and the dominant variant became alpha (>90 %), although an LMV was also present at important levels, having originated with the gain of mutation S494P, among others, and increased the virus’s affinity for human ACE2 [47]. The distribution of variants was better represented by SNPs than by next-generation sequencing (NGS). In contrast, however, since a non-random selection was made to perform NGS tests (only VOCs were chosen), variety was better determined by this method.

During the fourth wave in Asturias (June–August 2021), several variants circulated throughout Asturias, probably due to different sources of infection. This wave was more evident in younger people, that is those under 45 years old (data not shown), who had at this point not yet been vaccinated, and those returning from vacations outside the area. In other parts of the world gamma was the predominant, and almost exclusive, variant. However, WMV7 (VOC delta, lineage B.1.617.2), which had emerged in India in March 2021, later became dominant, and even displaced the rest of the variants worldwide. In June, beta appeared and alpha began to decrease until it was displaced by delta in August. In July 2021, five global and three regional variants were found around the world: none of them new. The ancestral variants also continued to circulate, so variants previously in circulation can gain, at any time and in any place, mutations that may affect the phenotype or the epidemiological characteristics of the virus, so continued monitoring is needed.

By analysing mutations at the global level, we lose those that have not yet dispersed worldwide and have only spread in specific areas or countries. Many countries have not sequenced enough virus samples for a full analysis to be carried out, and some countries have uploaded sequences to GISAID that were collected from single-source samples or zones of infection, meaning that the mutation pattern may be biased for a specific country or continent. When the mutations are reviewed country by country, however, we find dominant variants not included in the list of variants of interest. Additionally, this study employed non-synonymous mutations to understand the evolution and transmission of SARS-CoV-2. Because sample collection data may not reflect the actual infection data, analysis of the transmission path is only an approximation. Caution should be exercised with mutation analytics because the frequencies of the genotype groups may be unbalanced due to the unavailability of complete genomes in some countries and regions.

## Conclusions

SARS-CoV-2 reached Asturias as well as other parts of Spain and the rest of Europe, but early diagnosis and control kept the incidence during the first wave very low, even dropping to zero at some points. This ensured that few variants circulated. After summer 2020, however, the increase in the incidence of the virus around the world and the relaxation of social restrictions caused a second wave, and the alpha variant became dominant in Asturias from December 2020. At this point, when severe containment measures were not being imposed and only an incipient vaccination programme was in place, the epidemic reactivated in Asturias via the variants that were spreading throughout the world, although the region did not generate its own variants. In 2021, two further waves were observed in Asturias, both of which involved few variants from distant geographical areas, and, again, the region did not develop its own variant. The Asturian model indicates that the movement of people is capable of taking any variant to any location, but infection control measures (fast diagnosis, isolation, vaccination, etc.) are capable of controlling the circulation of these variants. Subsequently, the high vaccination coverage and the increase in diagnoses led to the transition to a strategy that monitors and directs actions to the most vulnerable people and areas, and in this situation new variants began to be found in Asturias [48]. Finally, use of the SNP method allowed us to determine, in more or less real time, the most important variants circulating, thus showing it to be a good method of epidemiological control complementary to WGS.

## Supplementary Data

Supplementary material 1

Supplementary material 2

Supplementary material 3

Supplementary material 4

Supplementary material 5

Supplementary material 6
