# Supplementary material for: Monitoring and tracking the spread of SARS-CoV-2 in Asturias, Spain
Source: Access Microbiol. 2023 Sep 27;5(9):000573.v4. doi: 10.1099/acmi.0.000573.v4 (PMC10569657; doi:10.1099/acmi.0.000573.v4)
Supplement: Supplementary material 6 [file acmi-5-573.v4-s001.pdf]

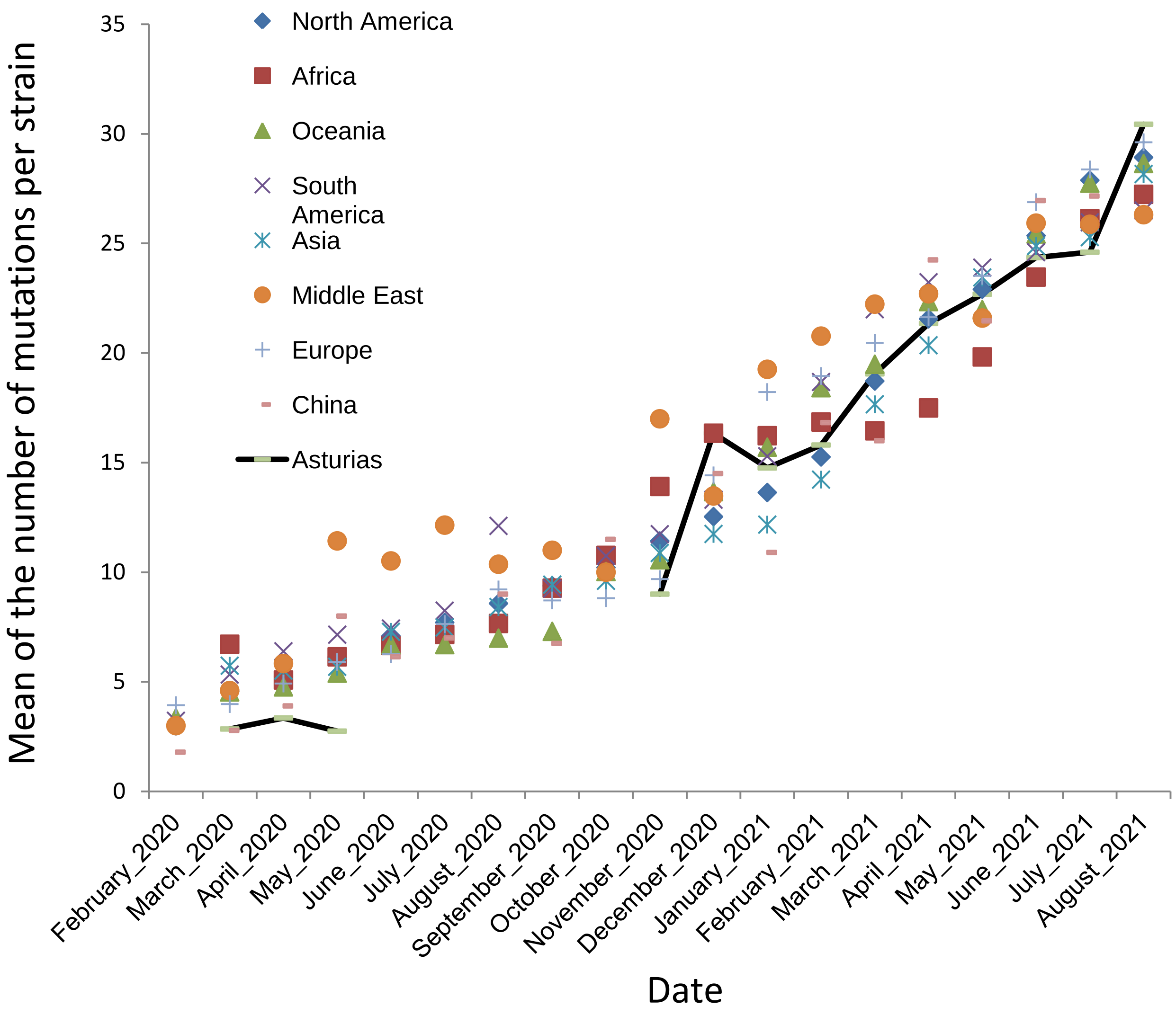

Fig S1. Mean of the number of mutations per strain and month in different places of the world.

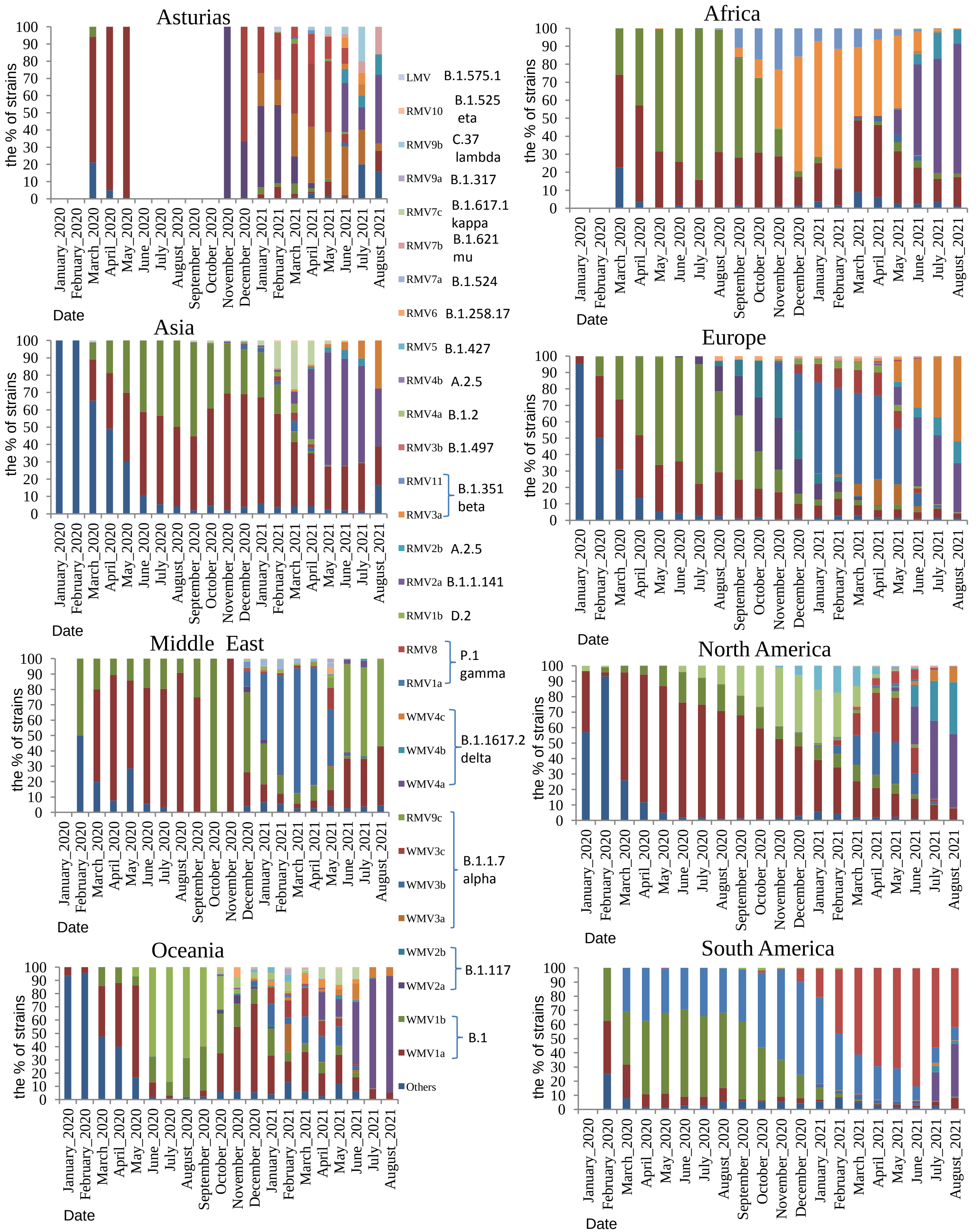

Fig S2. Distribution of the main variants in the geographical regions analyzed over time (Januay2020-August 2021). The x-axis indicates the % of strains in which the variant appears each month

SUPPLEMENTAL TABLE

Data Availability

GISAID Identifier: EPI_SET_230811vf
doi: 10.55876/gis8.230811vf
All genome sequences and associated metadata in this dataset are published in GISAID's EpiCoV database. To view the contributors of each individual sequence with details such as accession number, Virus name, Collection date, Originating Lab and Submitting Lab and the list of Authors, visit 10.55876/gis8.230811vf
Data Snapshot
EPI_SET_230811vf is composed of 3,091,151 individual genome sequences.
The collection dates range from 2010-12-06 to 2021-08-29;
Data were collected in 198 countries and territories;
All
